# Magnetic resonance imaging for clinical management of rectal cancer: Updated recommendations from the 2016 European Society of Gastrointestinal and Abdominal Radiology (ESGAR) consensus meeting

**DOI:** 10.1007/s00330-017-5026-2

**Published:** 2017-10-17

**Authors:** Regina G. H. Beets-Tan, Doenja M. J. Lambregts, Monique Maas, Shandra Bipat, Brunella Barbaro, Luís Curvo-Semedo, Helen M. Fenlon, Marc J. Gollub, Sofia Gourtsoyianni, Steve Halligan, Christine Hoeffel, Seung Ho Kim, Andrea Laghi, Andrea Maier, Søren R. Rafaelsen, Jaap Stoker, Stuart A. Taylor, Michael R. Torkzad, Lennart Blomqvist

**Affiliations:** 1 Department of Radiology, The Netherlands Cancer Institute, P.O. Box 90203, 1006 BE Amsterdam, The Netherlands; 2 Academic Medical Centre, Amsterdam, The Netherlands; 3 Catholic University School of Medicine, Rome, Italy; 4 Coimbra University Hospitals, Coimbra, Portugal; 5 Mater Misericordiae University Hospital, Dublin, Ireland; 6 Memorial Sloan-Kettering Cancer Center, New York, NY USA; 7 Guy’s & St Thomas’ NHS FT, London, UK; 8 Centre for Medical Imaging, University College London, London, UK; 9 Reims University Hospital, Reims, France; 10 Inje University Haeundae Paik Hospital, Busan, South Korea; 11 Sapienza - University of Rome, Rome, Italy; 12 Medical University of Vienna, Vienna, Austria; 13 Clinical Cancer Centre, Vejle Hospital, Vejle, Denmark; 14 Karolinska University Hospital and Karolinska Institutet, Stockholm, Sweden

**Keywords:** Magnetic resonance imaging, Cancer rectal neoplasms, Standards, Staging, Structured reporting

## Abstract

**Objectives:**

To update the 2012 ESGAR consensus guidelines on the acquisition, interpretation and reporting of magnetic resonance imaging (MRI) for clinical staging and restaging of rectal cancer.

**Methods:**

Fourteen abdominal imaging experts from the European Society of Gastrointestinal and Abdominal Radiology (ESGAR) participated in a consensus meeting, organised according to an adaptation of the RAND-UCLA Appropriateness Method. Two independent (non-voting) Chairs facilitated the meeting. 246 items were scored (comprising 229 items from the previous 2012 consensus and 17 additional items) and classified as ‘appropriate’ or ‘inappropriate’ (defined by ≥ 80 % consensus) or uncertain (defined by < 80 % consensus).

**Results:**

Consensus was reached for 226 (92 %) of items. From these recommendations regarding hardware, patient preparation, imaging sequences and acquisition, criteria for MR imaging evaluation and reporting structure were constructed. The main additions to the 2012 consensus include recommendations regarding use of diffusion-weighted imaging, criteria for nodal staging and a recommended structured report template.

**Conclusions:**

These updated expert consensus recommendations should be used as clinical guidelines for primary staging and restaging of rectal cancer using MRI.

***Key Points*:**

• *These guidelines present recommendations for staging and reporting of rectal cancer.*

• *The guidelines were constructed through consensus amongst 14 pelvic imaging experts.*

• *Consensus was reached by the experts for 92 % of the 246 items discussed.*

• *Practical guidelines for nodal staging are proposed.*

• *A structured reporting template is presented.*

**Electronic supplementary material:**

The online version of this article (10.1007/s00330-017-5026-2) contains supplementary material, which is available to authorized users.

## Introduction

In 2012 the European Society of Gastrointestinal and Abdominal Radiology (ESGAR) initiated an expert consensus meeting on magnetic resonance imaging (MRI) for the clinical management of rectal cancer, the results of which were published in European Radiology in 2013 [1]. To update these, 14 abdominal imaging experts from leading colorectal cancer centres participated in a formal consensus process aimed at defining a state-of-the-art MR protocol for rectal cancer and how imaging findings should be interpreted and reported. This update was precipitated by evolutions in the clinical management of rectal cancer since the 2012 guideline. Organ-preserving treatment strategies are used increasingly as alternatives to surgical resection in patients responding well to chemoradiotherapy (CRT) [2–4]. Accordingly, response assessment after chemoradiotherapy (CRT) is increasingly relevant and restaging MRI has become an intense topic for research. Moreover, functional MR imaging techniques such as diffusion-weighted imaging (DWI) and dynamic contrast-enhanced (DCE) MRI are increasingly incorporated into clinical MRI protocols. This 2016 update aimed to account for these recent developments. This paper reports findings from this consensus meeting and aims to provide up-to-date practice guidelines for MR imaging acquisition, interpretation and reporting for primary staging and restaging of rectal cancer.

## Materials and methods

### The consensus method

Similar to the methodology adopted for the 2012 consensus meeting [1] an adaptation of the RAND-UCLA Appropriateness Method (RAM) was chosen [5], which combines postal and face-to-face rounds. For the present update, the process can be summarised as follows:

Step 1 *Literature review*
  Two of the organising members (DL, MM) in consensus searched current literature to identify newly available indexed scientific evidence regarding rectal cancer imaging, published following the 2012 meeting, which was used to update the questionnaires used for the 2012 consensus meeting by addition of topics not discussed previously.
Step 2 *Update of the questionnaires*
  Updated questionnaires were constructed by two organising members (DL, MM), in consultation with two others (SB, RB). The original 2012 questionnaire comprised 236 items. Seventeen new items were added, which mainly concerned the current use of tumour node metastasis (TNM) staging systems [6, 7], the staging of tumours extending into the anal canal, criteria for nodal staging, use of structured reporting, and protocols for acquisition and evaluation of DWI. The questionnaire was divided into part A and part B. Part A included items reaching consensus in the 2012 meeting. Panellists were asked to indicate for each item whether they still agreed with the consensus statement reached previously or whether the item should be re-discussed. Part B combined items that did not reach consensus in 2012 with the additional 17 items derived from the updated literature review. All items were scored binomial (YES/NO; still valid or to be rediscussed) or ordinal (e.g. not recommended, recommended, mandatory), according to the individual item in question. Panellists were instructed to select ‘Mandatory’ for items that they considered were mandatory, ‘Recommended’ for items that they believed to be of additional benefit but that were not mandatory, and ‘Not recommended’ for items that they believed were not required and of no additional value.
Step 3 *Panel selection*
  The panel consisted of the same 14 panellists (BB, LC-S, HF, MG, SG, SH, CH, SHK, AL, AM, SR, JS, ST, MT) who participated in the 2012 consensus meeting. All were leading abdominal radiologists and members of ESGAR with recognised expertise and a publication track record within the field of rectal cancer imaging. The panel also included two non-voting Chairs (LB, RB) and three non-voting organising members (DL, MM, SB).
Step 4 *Questionnaire completion before the face-to-face meeting*
  Questionnaires were emailed to panellists on 11 May 2016. Panellists rated items independently with no interaction amongst each other and returned completed questionnaires by email.
Step 5 *Data analysis from questionnaire round*
  For each rated item from the electronic questionnaire round, two non-voting members (DL,MM) assessed whether or not consensus (defined as ≥ 80 % agreement) was reached.
Step 6 *Face-to-face panel meeting*
  A face-to-face panel meeting took place during the annual ESGAR meeting, Prague, 15 June 2016. Twelve of the 14 panellists attended. The meeting was moderated by two non-voting Chairs, RB and LB. Two non-contributing (non-voting) observers (DL, MM) documented key points of discussion and outcomes from the voting rounds. The results from the electronic questionnaire round formed the basis for discussion. Discussion included all items from the part A questionnaire selected for re-discussion by at least 20 % of the panellists in addition to all items from questionnaire part B that failed to reach consensus after the email round. Some items were rephrased or merged after face-to-face panel discussion (to reduce ambiguity or overlap) and as a result seven previously included items were discarded. After each item was discussed, panellists were asked to vote (using the same scoring systems as in the electronic round). Thirty items were not discussed face-to-face due to time constraints and were voted on by email subsequently.
Step 7 *Data analysis and reporting*
  Data from both electronic and face-to-face rounds were collected and descriptive metrics calculated by DL and MM. Each item was ultimately classified as: (1) ‘Appropriate’ with ≥ 80 % agreement, (2)’ Inappropriate’ with ≥ 80 % agreement or (3; Uncertain (no consensus, i.e. < 80 % agreement).

## Results

Demographic data of the panellists’ hospitals and MRI techniques are summarised in Table 1. A total of 246 items was voted on during the electronic and/or face-to-face rounds (236 items from the original 2012 questionnaires + 17 new items – 7 discarded/merged items). A complete overview of the results from the consensus procedure is provided in Appendix I in the Electronic Supplementary Material (ESM).

**Table 1:** Demographic data and MRI techniques of the panellists' base hospitals

Median (range) number of patients diagnosed with rectal cancer per year		110 (30–300)
MRI used as a standard staging technique for rectal cancer		100 %
Restaging after chemoradiation performed routinely		86 %
MR vendors*		
Siemens		57 %
Philips		21 %
GE		21 %
Unknown		29 %
Field strength		
1.5T		50 %
3.0T		7 %
1.5T and 3.0T		43 %
Use of a surface coil		100 %
Use of an endorectal coil		0 %
Routine use of spasmolytics (buscopan or glucagon)		43 %
Routine use of endorectal filling		29 %
Routine use of rectal (micro-)enema		14 %
Routine use of intravenous contrast material		29 %^
Diffusion-weighted MRI part of routine rectal MRI protocol		93 %
Dynamic contrast enhanced MRI part of routine rectal MRI protocol		29 %
Version of Tumour Node Metastases (TNM) staging system used		
TNM 5		7 %
TNM 6		0 %
TNM 7		86 %
unknown		7 %
Organ-preservation offered as a treatment option after chemoradiotherapy		
No		29 %
only local excision		21 %
only watchful waiting		7 %
both (local excision and watchful waiting)		43 %
MRI used as a follow-up modality (in centers performing organ preservation)		100 %

*21 % use multiple vendors

^ In the four centers routinely applying intravenous contrast it is used for DCE in 3/4 and for nodal staging in 1/4 cases

### Areas of consensus

**Fig. 1:**
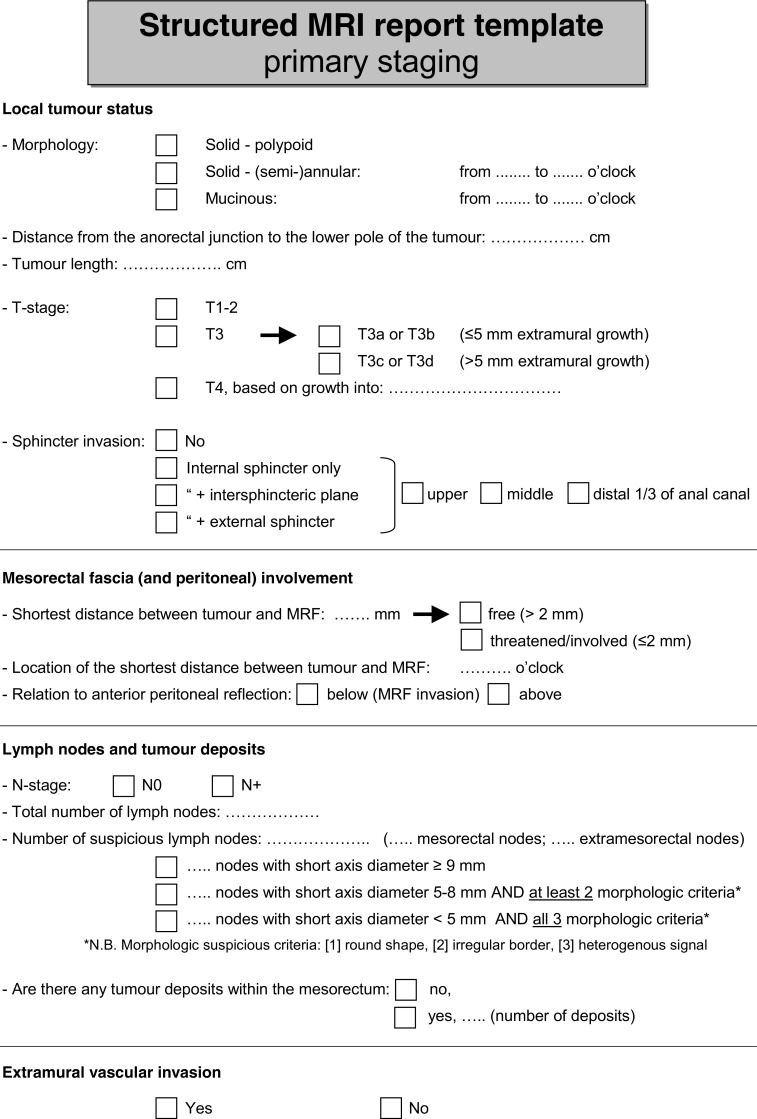

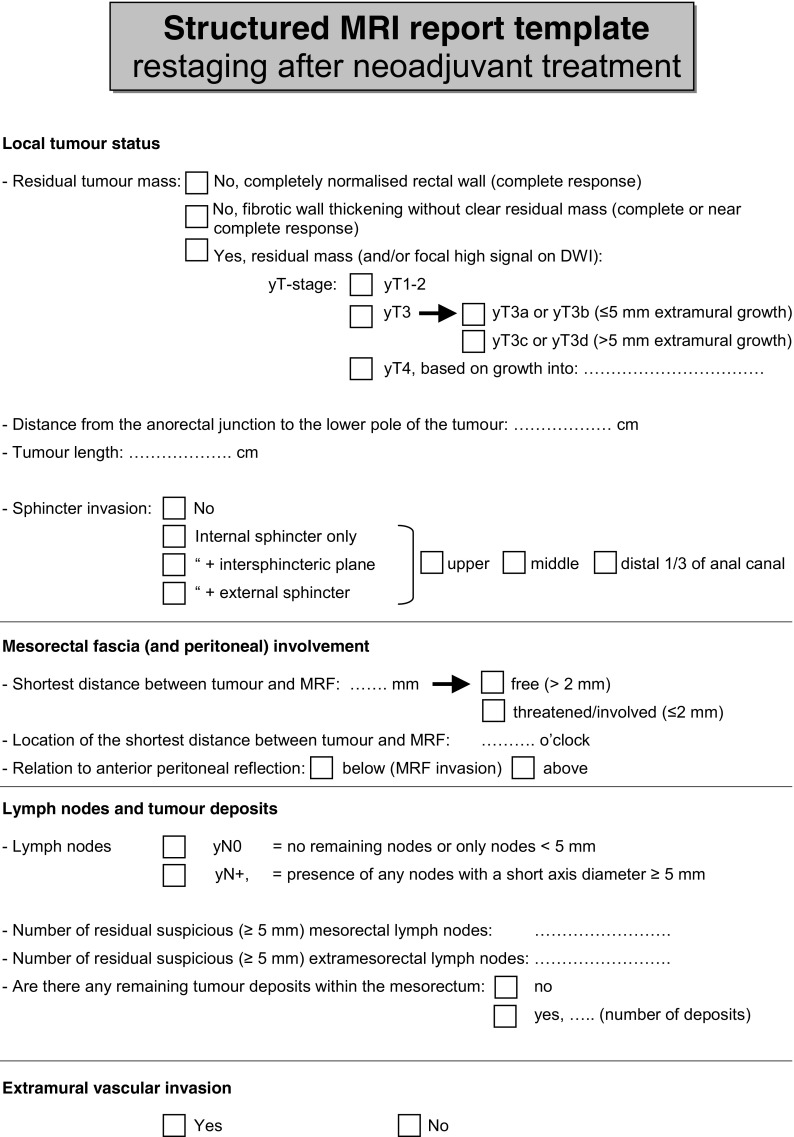
Template for structured MRI report for primary staging and restaging after chemoradiation

Consensus was reached for 226/246 (92 %) items. A summary of the recommendations based on decisions reached by the panel is given in Table 2. An overview of the main changes and additions to the 2012 consensus paper is provided in Table 3. In Table 4, advised criteria for nodal staging are provided. An example of a template for structured reporting as advised by the panel is given in Fig. 1.

**Table 2:** Synopsis and key recommendations (based on items for which ≥ 80 % consensus was reached)

I - Recommendations for MR image acquisition
a. hardware
MRI should routinely be performed for primary staging and restaging of rectal cancer
Endorectal ultrasound is the preferred technique for the differentiation and staging of T1 tumours
MRI should be performed with an external surface coil on a 1.5T or 3.0T MRI system
b. patient preparation
Use of an enema is not routinely recommended
(Use of spasmolytics may be useful to reduce bowel movement artefacts (no consensus: 57 % recommended/mandatory))
(Use of endorectal filling is not routinely advised (no consensus: 71 % not recommended))
c. sequences and sequence angulation
A routine protocol should (at least) include 2D T2-weighted sequences in 3 planes and a diffusion-weighted sequence (including at least a high b-value of ≥ 800)
Diffusion-weighted images (including Apparent Diffusien Coefficient maps) should mainly be assessed visually; quantitative ADC measurements are not routinely advised
Diffusion-weighted imaging is recommended for restaging of the yT-stage.
Fatsuppressed, T1-weighted (non-enhanced and contrast-enhanced) and dynamic contrast enhanced (DCE) sequences are not routinely recommended
Slice thickness (for the axial and coronal T2-weighted sequences) should be ≤ 3 mm
Transverse and coronal sequences should be angulated perpendicular and parallel to the rectal tumour axis, respectively.
In distal tumours a coronal sequence angulated parallel to the anal canal should be included to assess the relation between tumour and anal sphincter
II - Recommendations for MR image evaluation and reporting
a. primary staging
Structured reporting is recommended and should include the items described in the report template in Fig. 1
For nodal staging the criteria described in Table 4 are recommended
Stranding into the mesorectal fat is an equivocal sign that may indicate either a T2 or T3 tumour
The mesorectal fascia (MRF) is 'involved' if the distance between MRF and tumour is ≤1 mm
When a tumour shows stranding into the MRF, the MRF should be considered involved
A tumour that involves the MRF should be considered a T3 (and not a T4) tumour
Tumour invasion above the level of the peritoneal reflection (at the anterior side) should be considered at risk for peritoneal rather than MRF invasion
A tumour that invades the pelvic floor or pelvic side wall muscles should be considered a T4 tumour
A tumour that grows into the internal anal sphincter muscle should be considered a T3 (and not a T4) tumour
b. restaging after neoadjuvant treatment
Structured reporting is recommended and should include the items described in the report template in Fig. 1
For nodal restaging the criteria described in Table 4 are recommended
On T2-weighted MRI, a normalised, two-layered wall after CRT is suggestive of a complete response
On T2-weighted MRI, a completely hypointense (fibrotic) residue without an isointense mass indicates a complete or near-complete response
When considering organ preservation (watchful waiting) after CRT, MRI findings should be correlated with clinical examination (endoscopy / digital rectal examination)
If a fatpad re-appears between the tumour and MRF after CRT, the MRF should be considered uninvolved/cleared.
Persistent stranding of tumour into the MRF should be considered an equivocal sign that may or may not indicate persistent MRF involvement
III - MRI performance
a. T2-weighted MRI
Primary staging
2D T2-weighted MRI can be used to reliably (≥80 % accurate):
Differentiate between T2 and T3 tumours
Differentiate between non-involved and involved mesorectal fascia
2D T2-weighted MRI is not accurate to differentiate between T1 and T2 tumours
b. Diffusion-weighted MRI
Primary staging
Diffusion-weighted MRI is not accurate to:
differentiate between T1 and T2 tumours
differentiate between T2 and T3 tumours
differentiate between N0 and N+ stage
differentiate between non-involved and involved mesorectal fascia
assess EMVI
Restaging
Diffusion-weighted MRI is not accurate to:
differentiate between T1 and T2 tumours
differentiate between N0 and N+ stage
assess EMVI

**Table 3:** Main changes and additions to the 2012 consensus meeting results

Main changes
Slice thickness should be ≤ 3 mm (recommended slice thickness ≤ 4 mm in 2012)
The circumferential location of the tumour within the rectal wall (e.g. from X to X o'clock) should routinely be reported (no consensus in 2012)
EMVI should routinely be reported at primary staging as well as at restaging after CRT (no consensus on reporting of EMVI after CRT in 2012)
Main additions
Recommendation to report T3 substages (T3a,b,c,d) and T4 substages (T4a,b)
Recommendation to perform structured reporting (example of structured reporting template is provided in Fig. 1)
Specified criteria for nodal staging (see Table 4)
Specified definitions for T3 versus T4 tumours in case of sphincter invasion, MRF, pelvic wall/floor involvement and for tumours below or at/above the level of the anterior peritoneal reflection
Specified indications for DWI
Specified minimal requirements for DWI (protocol should include at least a high b-value of ≥ b800; assessment should consist of visual evaluation of DW images and ADC-map, quantification of ADC is not routinely recommended)

**Table 4:** Practical guidelines for nodal staging

Primary staging
Criteria for malignant node:
1. Short axis diameter ≥ 9 mm
2. Short axis diameter 5–8 mm AND ≥ 2 morphologically suspicious characteristics*
3. Short axis diameter < 5 mm AND 3 morphologically suspicous characteristics*
4. All mucinous lymph nodes (any size)
* Morphologically suspicious criteria:
Round shape
Irregular border
Heterogeneous signal
Restaging (after long course neoadjuvant treatment + downstaging interval)
All nodes with a short axis diameter < 5 mm should be considered benign
For nodes with a short axis diameter ≥ 5 mm no reliable criteria exist.
As a practical guideline these nodes should be considered malignant.

NB. These criteria should only be applied using high-resolution (≤ 3 mm slice thickness) transverse images.

NB2. These criteria specifically apply to nodes within the mesorectal compartment, but may also be adopted for other regional extramesorectal (i.e. obturator and iliac) nodes

NB3. The criteria described above are intended as a practical guideline. The panel acknowledges known inaccuracies of MRI for nodal staging.

### Areas lacking consensus

In 20/246 (8 %) items no consensus was reached by the panel. The items that did not reach consensus are listed in Table 5. Main items were patient preparation, use and performance of diffusion-weighted MRI and the diagnostic performance of T2-weighted MRI in the restaging setting.

**Table 5:** Items lacking consensus

Patient preparation
Use of spasmolytics (recommended by 57 % of the panel)
Use of endorectal filling (recommended by 29 % of the panel)
Diffusion-weighted imaging
There was no consensus on the number of b-values required (57 % consensus at least 2; 43 % consensus at least 3)
There was no consensus on whether DWI should be used for:
assessment of T-stage at primary staging (31 % not recommended; 46 % unsure*; 8 % recommended; 15 % mandatory)
assessment of N-stage at primary staging (23 % not recommended; 38 % unsure*; 31 % recommended; 8 % mandatory)
assessment of yN-stage at restaging (15 % not recommended; 31 % unsure*; 23 % recommended; 23 % mandatory)
assessment of MRF at restaging (15 % not recommended; 62 % unsure*; 15 % recommended; 8 % mandatory)
MRI performance
There was no consensus whether 2D T2-weighted MRI is reliable (≥ 80 % accurate) to:
differentiate between N0 and N+ stage at primary staging (not reliable with 69 % consensus)
assess EMVI at primary staging (reliable with 69 % consensus)
differentiate between a complete response and residual tumour at restaging (not reliable with 69 % consensus)
differentiate between yT1-2 and yT3-4 tumours at restaging after CRT (reliable with 62 % consensus)
differentiate between yN0 and yN+ stage at restaging after CRT (reliable with 62 % consensus)
differentiate between non-involved and involved MRF at restaging after CRT (reliable with 62 % consensus)
assess EMVI at restaging after CRT (reliable with 54 % consensus)
There was no consensus whether diffusion-weighted MRI is reliable (≥ 80 % accurate) to:
differentiate between a complete response and residual tumour at restaging (reliable with 54 % consensus)
differentiate between yT1-2 and yT3-4 tumours at restaging after CRT (not reliable with 69 % consensus)
differentiate between non-involved and involved MRF at restaging after CRT (not reliable with 69 % consensus)
MRI reporting
reporting of N-substages (N1a, N1b) (recommended by 31 % of the panel)
There was no consensus whether a tumour that invades the external anal sphincter should be considered a T3 (29 % consensus) or T4 (71 % consensus) tumour

*Unsure indicates that it is not routinely recommended, but may be useful for particular cases

The panel ultimately achieved consensus for 92% of the individual items discussed, which is comparable to outcomes from the 2012 meeting where the figure was 88 % [1]. Although consensus was not achieved in 20/246 (8 %) items, reasonable agreement (> 60 %) was still reached in 14 out of these 20 items.

### Imaging techniques

Consistent with the 2012 results, the panel reached consensus that MR imaging is mandatory and the technique of first choice for both primary staging and restaging of rectal cancer, with the exception of staging for early tumours considered for local excision. In these cases patients should be referred for (additional) endorectal ultrasound (EUS), given its superior diagnostic performance for differentiating T1 from T2 tumours [8, 9]. Additionally, the panel agreed that when considering organ-preservation (‘watchful waiting’) as a treatment option after chemoradiotherapy, it is mandatory to correlate the restaging MRI findings with clinical examination (digital rectal examination and endoscopy) as this combination has been shown to be most accurate when establishing a correct diagnosis of complete response [10]. The best timing to perform the restaging MRI (i.e. how many weeks after completion of CRT) is an issue of ongoing debate, but there is evidence suggesting that longer waiting intervals may increase response rates [11, 12]. In recently published reports that routinely included MRI to select patients for organ-preservation, a waiting interval of around 8 weeks was typically employed [2, 3].

### Patient preparation

As for 2012, no consensus was reached regarding the use of spasmolytics (e.g. Buscopan® or Glucagon) or endorectal filling. Routine spasmolytics were advised by 57 % of the panel (compared to 50 % in 2012) and endorectal filling by 29 % (17 % in 2012). Panellists that apply spasmolytics suggested that it can be particularly beneficial for upper rectal tumours and when imaging is performed at 3.0T, because in these cases bowel movement artefacts are most prevalent. Regarding endorectal filling, the panel noted that it can be useful in specific cases, particularly to reduce susceptibility artefacts related to luminal gas during diffusion-weighted MRI. The increased use of DWI in current practice may therefore explain the increased recommendation for endorectal filling by the panel compared to 2012. However, its use was not recommended routinely, mainly because rectal wall distension may interfere with interpretation of the distance between the tumour and the mesorectal fascia [13]. When choosing to fil the rectum nonetheless, the panel therefore suggests using a volume of only approximately 60 ml of gel, since higher volumes will compress perirectal tissues significantly [14]. Another potential drawback of rectal gel filling is that the high T2 signal of the gel may cause T2 shine through effects on DWI. A potential alternative currently being investigated is the use of a micro-enema to reduce the amount of luminal gas. This option will need further exploration as results are presently only single centre [15, 16].

### Criteria for MR imaging assessment

The 2012 meeting reached consensus for the majority of imaging criteria discussed. The majority of debate revolved around which criteria should be used for nodal staging. In 2012 the panel agreed that no single size threshold was sufficiently accurate to differentiate benign from metastatic nodes. Furthermore, it was agreed with 70–75 % consensus that additional morphological criteria (shape, border, signal heterogeneity) can be beneficial to help characterisation. The 2016 panel agreed that, despite the fact that nodal staging remains challenging with well-known inaccuracies, it would be desirable to at least offer a practical guideline incorporating both size and morphology. This resulted in the criteria described in Table 4, which were based on criteria described in Dutch evidence-based guidelines on rectal cancer treatment [17]. The main aim of this approach was to establish more stringent criteria and thereby increase the threshold so as to avoid over-calling nodes as malignant. The literature suggests around 25 % of nodes are overstaged [8], which could mean unnecessary preoperative treatment and associated short-term (e.g. proctitis) and long-term (e.g. faecal incontinence, bowel and urogenital dysfunction) morbidity [18]. It has even been suggested that nodal staging by imaging should be discarded altogether, because of known staging inaccuracies and because it has been suggested that patients with ‘good prognostic’ tumours (defined on MRI as ≤cT3b stage without MRF involvement) have good outcomes regarding survival and local recurrence rates, irrespective of nodal stage [19]. However, this strategy has not been adopted widely and nodal stage as determined by imaging, despite proven inaccuracies, is still believed to be an important treatment determinant and included in most current guidelines [18–20]. Another point of debate was whether or not the criteria described in Table 4 are also applicable to extramesorectal (obturator and iliac) nodes. There is to date no solid evidence regarding specific or alternative (size) criteria for extramesorectal nodes and as such it was not deemed feasible to recommend any specific criteria for these nodes. Therefore the panel agreed that, from a practical point of view, the same criteria recommended for the mesorectal lymph nodes (Table 4) may for now also be used for these extramesorectal nodal stations.

Another addition to the 2012 guideline was the use of T-substages such as T3a,b,c,d (which categorise tumours via differing depths of extramural invasion). Also, discussion around the definition of T3 versus T4 stage in various settings was added. The problem with these definitions is that they can depend on the TNM version used, which may vary between and even within centres, precipitating confusion. For example, the panel could not agree unanimously (93 % consensus) whether a tumour invading the mesorectal fascia was T3 or T4. Similarly, the panel could not reach consensus whether a tumour involving the external anal sphincter should be T3 (29 % of the panel) or T4 (71 %). To avoid confusion, the panel agreed that, regardless of the TNM version employed, the main aim should be to provide the clinician with an MR report that includes and describes clearly all relevant information needed for treatment planning, rather than to focus on terminology. For example, for low tumours involving the anal sphincter complex, it is pertinent to describe: (a) whether the tumour invades only the internal sphincter muscle or also involves the intersphincteric plane and external sphincter, (b) whether sphincter invasion involves only the proximal one-third of the complex or extends into the middle and/or lower third, and (c) whether the pelvic floor (levator) is involved. Such information is pivotal when deciding the surgical approach, e.g. whether or not sphincter-saving resection is feasible without compromising local control. In such cases, accurate anatomical description of local extent provides more relevant information than the T-stage alone.

### MRI reporting

In addition to the MR imaging criteria described above, the panel agreed that description of tumour location in relation to the anterior peritoneal reflection should be reported as an extra item (omitted from the 2012 guidelines). This item was added as it can be a potential source of confusion: The anterior peritoneal reflection is a landmark that is usually recognised easily on MRI and separates the intra- and extra-peritoneal portions of the mesorectal compartment [20]. Above the anterior peritoneal reflection, the mesorectal compartment is no longer enveloped by the mesorectal fascia on its anterior aspect. As such, anterior mesorectal fascia involvement should only be reported when below the level of the anterior peritoneal reflection.

Another important addition to the 2012 guideline is a recommendation for structured reporting. A structured report template has been suggested previously and published online by the Radiological Society of North America (RSNA; radreport.org). In Fig. 1 we propose an alternative template (both for primary staging and for restaging after neoadjuvant treatment) based on the specific recommendations arising from this 2016 updated consensus meeting. Studies have shown that implementation of structured report templates can improve the quality of MRI reporting for rectal cancer staging compared to free-text formats, and leads to higher satisfaction levels from referring surgeons [21, 22]. Accordingly, structured reporting is now recommended by the panel unanimously. The structured report (see Fig. 1) includes measurement of tumour longitudinal extent. This, however, was an item that was debated face-to-face. Although the panel agreed unanimously that ‘some measure of tumour size’ should be reported, there was no clear consensus on a specific metric, i.e. whether this should be one-dimensional, three-dimensional or a volume measurement, and if and how after CRT an estimation of the tumour volume reduction should be provided. There is no solid evidence that favours one over another, although some authors have suggested that, specifically for assessment of chemoradiotherapeutic response, whole volume measurements may be preferable [23]. The panel acknowledges that several options exist but from a practical point of view decided to include tumour length as the main metric in the structured report template in Fig. 1, as this was deemed to be most commonly used and more practically applicable than other metrics, with good reported measurement reproducibility [20, 23, 24].

Finally, the panel agreed that assessment of extramural vascular (or venous) invasion (EMVI) should be reported routinely, both for primary staging as well as for restaging after CRT. In 2012, reporting of EMVI after CRT was not advised unanimously. This change is likely the result of emerging evidence supporting EMVI as an important prognostic staging factor [25–27].

### Diffusion-weighted imaging and dynamic contrast-enhanced MRI

Since publication of the previous ESGAR consensus guidelines in 2013, numerous reports have emerged investigating use of functional imaging techniques for rectal cancer (re)staging, of which DWI and dynamic contrast-enhanced MRI have been researched most extensively. As such, the panel decided to specifically address whether these techniques should be incorporated into routine protocols and, if so, in which circumstances. In concordance with the 2012 recommendations, the panel agreed that diffusion-weighted MRI should be performed routinely, in particular for restaging to evaluate response (the yT-stage) to chemoradiotherapy. This decision reflects the increasing volume of data reporting that DWI can improve the performance of MRI for T-restaging after neoadjuvant treatment, specifically for differentiation between complete and partial response [28–32]. Although 85 % of the panel recommended using DWI to assess yT-stage after CRT, only 54 % believed that DWI alone is able to identify patients with complete response reliably. The panel recognises that findings from both T2-weighted MRI and DWI will need to be combined with digital rectal examination and endoscopy to obtain the most accurate diagnosis when aiming to identify complete responders for organ-preserving treatment strategies [10]. For all other indications (primary T-staging, (y)N-staging, assessment of MRF involvement and EMVI) the panel either reached consensus or the majority of panellists agreed (achieving 54–77 % consensus) that there is no clear added benefit for DWI based on current evidence, although several panellists did point out that DWI may be of value for individual cases. Furthermore, the panel agreed unanimously that diffusion-weighted images (and ADC maps) should be assessed qualitatively, with no current role for quantification of ADC in daily practice due to a lack of standardised protocols and validated thresholds. Regarding DCE-MRI, the panel reached full consensus that, although some promising data are available [33–37], it should currently be considered as a research tool and not be adopted routinely. Nevertheless, panellists again acknowledged that DCE may be useful for individual cases, particularly to improve tumour conspicuity after neoadjuvant treatment and for assessment of mucinous tumours.

#### Methodological limitations

Our consensus process has some limitations. Two panellists were absent during the face-to-face meeting. For items discussed and voted upon during this meeting, we therefore calculated the percentage of agreement based on those panellists present. As for the 2012 version, our recommendations concern predominantly the staging of non-mucinous adenocarcinomas. No specific recommendations regarding assessment of mucinous tumours (except for mucinous lymph nodes described in Table 4) were made. Although unavoidable for face-to-face methodology, bias might be introduced by undue influence from those panellists with ‘dominant’ personalities. We attempted to counter this via non-voting Chairs who ensured that discussions were well-balanced. Finally, the panel is selected and thus may not be fully representative of all opinions.

## Electronic supplementary material

ESM 1 (XLS 74 kb)
